# Salivary Diagnostics in Pediatrics and the Status of Saliva-Based Biosensors

**DOI:** 10.3390/bios13020206

**Published:** 2023-01-30

**Authors:** Hayeon Min, Sophie Zhu, Lydia Safi, Munzer Alkourdi, Bich Hong Nguyen, Akshaya Upadhyay, Simon D. Tran

**Affiliations:** 1 McGill Craniofacial Tissue Engineering and Stem Cells Laboratory, Faculty of Dental Medicine and Oral Health Science, McGill University, 3640 University Street, Montreal, QC H3A 0C7, Canada; 2 CHU Sainte Justine Hospital, Montreal, QC H3T 1C5, Canada

**Keywords:** biosensors, biomaterials, oral diagnostics, pediatric population, salivary diagnostics, salivary biomarkers

## Abstract

Salivary biomarkers are increasingly being used as an alternative to diagnose and monitor the progression of various diseases due to their ease of use, on site application, non-invasiveness, and most likely improved patient compliance. Here, we highlight the role of salivary biosensors in the general population, followed by the application of saliva as a diagnostic tool in the pediatric population. We searched the literature for pediatric applications of salivary biomarkers, more specifically, in children from 0 to 18 years old. The use of those biomarkers spans autoimmune, developmental disorders, oncology, neuropsychiatry, respiratory illnesses, gastrointestinal disorders, and oral diseases. Four major applications of salivary proteins as biomarkers are: (1) dental health (caries, stress from orthodontic appliances, and gingivitis); (2) gastrointestinal conditions (eosinophilic esophagitis, acid reflux, appendicitis); (3) metabolic conditions (obesity, diabetes); and (4) respiratory conditions (asthma, allergic rhinitis, small airway inflammation, pneumonia). Genomics, metabolomics, microbiomics, proteomics, and transcriptomics, are various other classifications for biosensing based on the type of biomarkers used and reviewed here. Lastly, we describe the recent advances in pediatric biosensing applications using saliva. This work guides scientists in fabricating saliva-based biosensors by comprehensively overviewing the potential markers and techniques that can be employed.

## 1. Introduction

**Figure 1:**
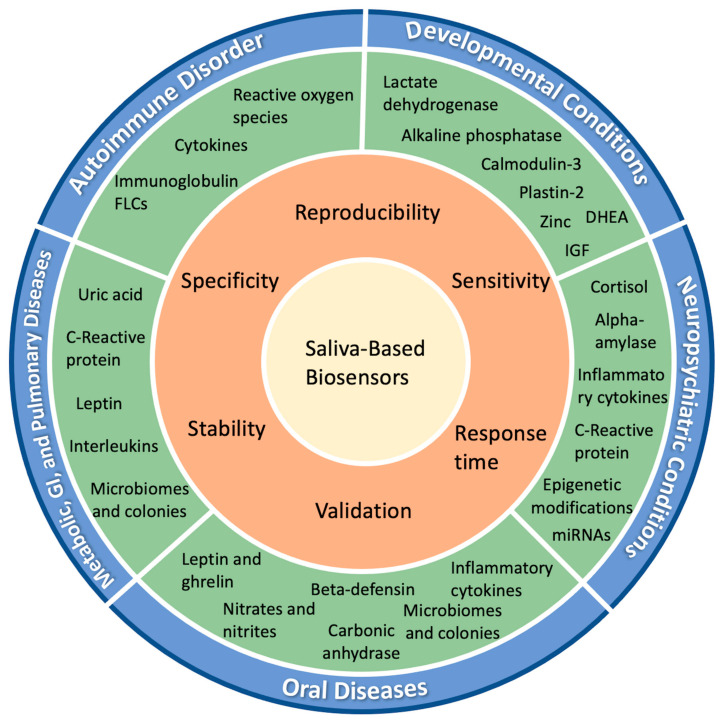
Prospects for saliva-based biosensors: various biomarkers have been identified in systemic conditions; discussed here are autoimmune disorders, developmental conditions, neuropsychiatric conditions, metabolic disorders, gastrointestinal disorders, pulmonary diseases, and oral diseases. For biosensing applications, they should be assessed for: their reproducibility at different time points, locations, and populations; specificity towards the marker and disease; sensitivity, meaning they should be able to detect as low a concentration of the markers as possible, ideally lower than a clinically available test; the response time of the assay, preferably shorter for quicker results; and validation in clinical conditions in larger population sizes.

Currently, blood and urine tests are commonly carried out to assess one’s immune status, neuropsychiatric state, and development status. However, invasive screening, diagnostic, and prognostic tests induce a high-stress level in the pediatric population and their parents. Recent research has shown that salivary biomarkers can be an alternative non-invasive test, particularly useful in reducing stress in children between 0 and 18. This study aims to screen the current research on the use of salivary biomarkers in diagnosing autoimmune diseases, developmental disorders, and cancer, as well as neuropsychiatric, pulmonary, gastrointestinal, and oral diseases. It is an effort to highlight the role of saliva as a sample for its utilization in biosensing applications (Figure 1).

## 2. Materials and Methods

### 2.1. Study Design

This study involved an Ovid database search with the following search strategy: (1) saliva.mp. (69,706 articles); (2) salivary diagnostics.mp. (172 articles); (3) saliva*.mp. (131,830 articles); (4) oral.mp. (767,765 articles); (5) 1 or 2 or 3 or 4 (876,003 articles); (6) biomarkers.mp. or Biomarkers, Tumor/or Biomarkers/or Biomarkers, Pharmacological/(639,768 articles); (7) 5 and 6 (24,223 articles); (8) pediatrics.mp. or Pediatrics/or Child/(1,892,565 articles); (9) 7 and 8 (1285 articles).

### 2.2. Study Participants

The publication year was limited to “2015 to current”, resulting in a total of 698 articles that fit the criteria above at the time of preparing this manuscript in August 2022. We established inclusion and exclusion criteria for this study to filter those articles further. Inclusion criteria: the research paper must focus on using salivary biomarkers to diagnose a specific disease or disorder in the pediatric population, between 0 years old and 18 years old. Exclusion criteria: review articles and case reports were excluded.

### 2.3. Data Collection

We selected 161 articles and divided them into 7 sections: pulmonary and gastrointestinal diseases; developmental disorders; infectious diseases; neuropsychiatric diseases; oral diseases; oncology; and autoimmune diseases. There were: 9 articles related to autoimmune diseases; 27 articles for developmental disorders; 7 articles for infectious diseases; 57 articles for neuropsychiatric diseases; 27 articles for oral diseases; 3 articles for oncology; and 31 for pulmonary and gastrointestinal diseases. Each section is further divided into 5 specific applications of salivary biomarkers: metabolomics, proteomics, genomics, transcriptomics, and microbiomics.

## 3. Salivary Biomarkers as Diagnostic Tools in Autoimmune Disorders

### 3.1. Metabolomics

A study by Aral et al. investigated the salivary oxidative status of children with Type I Diabetes Mellitus (T1DM) and its implications regarding the risk of periodontal disease (PD) development. The authors observed that, when compared to their healthy counterparts, children with T1DM have a greater Oxidative Stress Index (OSI) (*p* < 0.05) [1]. To explain this finding, the authors observe that chronic hyperglycemia impairs reactive oxygen species (ROS) elimination. The elevated ROS produces tissue damage, thus contributing to the development of PD. This finding highlights the potential of salivary OSI as a prognostic tool for PD in children with T1DM, which would enable physicians to intervene earlier to improve the course of the patient’s periodontal disease and, by extension, the duration of their diabetes.

### 3.2. Proteomics

A study by Collin et al. compared the salivary levels of several cytokines in children with Juvenile Idiopathic Arthritis (JIA) to those in healthy children. While IL-8 levels initially seemed to be higher in children with JIA, the authors ultimately concluded that there was no significant difference in the level of any investigated cytokines across the two group [2]. To explain this finding, the authors highlight that the children with JIA were taking immunosuppressive therapy throughout the study, which could hold their immune activity, and thus salivary cytokine levels, in check. Despite this, it could be possible to create a prognostic tool for children with JIA based on salivary cytokine levels. Such a tool would help monitor the children’s response to therapy and the prognosis of their symptoms.

Immunoglobulin-free light chains (FLCs) were identified as a strong predictor in the prognosis of Pediatric-Onset Multiple Sclerosis (POMS), and its relapse [3]. Detecting the higher levels of free light chains early can potentially enable physicians to initiate stronger therapy to reduce the severity of the children’s symptoms. Additionally, compared to current prognostic modalities, saliva collection is significantly less invasive than a lumbar puncture; it avoids gadolinium’s potentially harmful deposition in the brain from repeated MRIs.

In a similar fashion to the studies above, Gomez Hernandez et al. investigated the saliva of children with Sjogren’s Syndrome (SS) for cytokines, chemokines, and biomarkers (CCBM) that can serve as a diagnostic instrument for the condition [4]. The authors found 43 CCBM levels significantly elevated in children with SS, compared to healthy controls. Judging that number is too high for practical use; the authors recommend a test based on IL-27 and CCL-4 alone, or a quintuple test of IL-27, MIA, CCL-4, TNFRSF18 and TNFA, to aid in diagnosing SS. The authors encourage the pursuit of further studies to link these CCBM to Sjogren’s, as identifying such salivary biomarkers, which would appear early before the onset of symptoms, would enable physicians to intervene early and avert the course of the disease. A common limitation of the studies reviewed here is the small sample size. Indeed, the authors of the above papers invite further research to consolidate their findings. Further, other markers pertaining to transcriptomics and genomics can be explored for such conditions (Table 1).

## 4. Salivary Biomarkers as Diagnostic Tools in Developmental Conditions

### 4.1. Metabolomics

Bone-specific alkaline phosphatase (B-ALP) is a bone formation marker whose levels fluctuate with puberty. Al-Khatieeb et al. hypothesized that the level of ALP and LDH could affect the compliance of patients to monoblock therapy for orthoskeletal treatment. A high level of these enzymes was observed but was not significant after performing statistical analysis [5]. In another study, ALP, protein concentration, and chronological age with cervical vertebral maturation stages (CVMS) were used as non-invasive biomarkers to determine skeletal maturity. Salivary levels of ALP in conjunction with chronological age were accurate predictors for CVMS in 53.2% of cases [6]. ALP levels were co-related with variable stages of skeletal maturity (*p* = 0.0003) [7].

The early diagnosis and intervention of those with Autism Spectrum Disorder (ASD) have been linked with enhanced functional outcomes. The diagnosis, intervention, and prognosis would be facilitated by biomarker sampling, particularly salivary zinc concentrations. Low salivary zinc in those with ASD may contribute to the pathogenesis of autism [8]. Salivary metabolites were found to be higher in children experiencing pain when compared to those not experiencing pain. Collecting these non-invasive biomarkers may contribute to future pain management interventions in children with ASD [9].

### 4.2. Proteomics

It was found that insulin-like growth factor (IGF-1) and insulin-like growth factor binding protein-3 (IGFBP-3) may be used to estimate skeletal maturity. It was found that low salivary IGF-1 levels were present during puberty and lowered as pubertal growth ended. As a result, these salivary levels may be used to predict the completion of skeletal growth and may help predict when children would be most amenable to orthodontic treatments [10,11].

Increased levels of salivary proinflammatory markers, such as interleukin-6 (IL-6) and c-reactive protein (CRP), have been associated with child maltreatment. IL-6 secretions have been found to follow a clear circadian rhythm, suggesting that a disrupted rhythm is indicative of a dysfunctional inflammatory system. These findings suggest that a disrupted IL-6 rhythm, and, as a result, a disrupted inflammatory system, may be linked with childhood traumas (*p* = 0.031) [12]. Mucosal secretory immunoglobulin A (s-IgA) plays a critical role in the immune system. It has been found that acute psychosocial stress stimulates s-IgA secretion in adults and children who have undergone maltreatment. This is suggestive of child maltreatment prematurely aging their immune system [13]. Familial social and economic instability in early childhood has been linked to immune system dysregulation, as is evidenced by DNA shedding of the Epstein-Barr virus (EBV) salivary biomarkers. Traumatic early childhood events resulted in a 100% increase in EBV DNA shedding among previously infected adolescents. This indicates that the salivary biomarkers of EBV may be indicative of childhood trauma [14].

The protein composition of saliva has been studied from early on to find associated markers for the diagnosis and prognosis of ASD [15,16]. The mass spectrometric analysis of a patient cohort of 132 proteins were increased in ASD-positive patients, 25 of which were associated with a severe to moderate group of the elevated proteins, suggested as biomarkers for ASD calmodulin-3, plastin-2, and protein s100-a7 [17].

### 4.3. Genomics

Those with Cleft Lip Palate (CLP), one of the most common congenital malformations of the head and neck, are found to be more likely to present with accompanying anomalies including Defects of Enamel (DDE). Matrix Metallopeptidase 2 (MMP2), a membrane-bound protein containing collagen-degrading capabilities, plays a crucial role in tooth formation and mineralization. The findings suggest that MMP2 may play a role in a genomic approach to establish any potential risks for those with CLP [18]. Supernumerary teeth are one of the most common dental anomalies seen in the pediatric population. An immunohistochemistry panel showed that those with supernumerary teeth presented with an enhanced expression of Wingless (Wnt) and Sonic Hedgehog (SHH) proteins, both of which are pro-tumorigenic proteins [19].

It has been shown that psychological stress elicits telomere shortening. Through salivary samples via PCR testing, it was found that families with an ASD child endure exceeding levels of psychological stress [20]. Using these biomarkers may contribute to the early diagnosis of any psychological stress and respective intervention, which would optimize mental well-being and the quality of life. Maternal stress affects an infant’s neurocognitive development before, during, and post-pregnancy. Certain CpGs were found to be linked genetically, including YAP1, TOMM20, and CSMD1.

Additionally, two differentiated methylation groups (DMR), DAXX and ARL4D, were found to be related to maternal stress measures. An early assessment of maternal stress may contribute to preventative health measures for the infant, further optimizing their health and the quality of life [21]. Upon examining polymorphisms of the STS and SULT2A1 genes, dehydroepiandrosterone (DHEA) and its sulfated form, DHEA-A, and their relationship to Attention Deficit Hyperactivity Disorder (ADHD), it was found that the levels of DHEA were positive correlated with attention. This may highlight the potential role of ADHD and its pathogenesis with regard to STS polymorphisms and neurosteroid levels [22] (Table 2).

### 4.4. Transcriptomics

Circulating microRNA (miRNA) was compared to cerebrospinal fluid miRNA levels when assessing miRNA as a biomarker for pediatric concussions. Six miRNAs had similar changes in both CSF and salivary levels, including miR-182-5p, miR-221-3p, mir-26b-5p, miR-320c, miR-29c-3p, and miR-30e-5p. More specifically, concentrations of miR-320c were found to be directly correlated with attention deficit. This suggests that salivary miRNA may be an accurate potential biomarker for traumatic brain injuries [25].

Several miRNAs have been found to potentially be used as biomarkers in diagnosing and treating those with ASD. The most accurate miRNAs to differentiate between typically developing children and those with ASD were miR-23a-3p, miR-32-5p, and miR-628-5p [24].

### 4.5. Microbiomics

ASD has been linked with many comorbidities, including gastrointestinal abnormalities, dental disease, and allergies. Oral and gut microbiomes are suggested to play important roles in inflammation and immune dysfunction pathogenesis. More specifically, 16S rRNA gene amplicon sequencing helps distinguish the oral and gut macrobiotics of patients with ASD. Altering the oral and gut microbiome may potentially address and ameliorate co-morbidities associated with those with ASD [23].

## 5. Salivary Biomarkers as Diagnostic Tools in Neuropsychiatry

### 5.1. Metabolomics

A plethora of evidence supports that measuring salivary cortisol and alpha-amylase levels serves as stress biomarkers. The application of salivary stress biomarkers spans from pediatric dental anxiety to internalizing or externalizing disorders. The partial hypofunction of hypothalamus-pituitary-adrenal (HPA) was found to be associated with pediatric patients suffering from ADHD by assessing the cortisol levels to induced stress, which was found to be reduced in positive patients (*p* = 0.034) [26]. In addition, salivary immune biomarker secretory immunoglobin A (s-IgA) is often measured alongside HPA biomarkers to explore the interactions between stress, the HPA axis, and the immune system [27]. Obtaining samples through saliva is a great alternative to blood collection in the pediatric population, especially for children with cerebral palsy. In patients with cerebral palsy, the sympathetic nervous system predominates, causing vasoconstriction and making peripheral venous blood more difficult to access. A study demonstrated, with non-invasive salivary diagnostics, that higher levels of inflammatory markers in patients with cerebral palsy are associated with excessive constipation and a reduced quality of life [28].

While using salivary stress biomarkers, such as cortisol and alpha-amylase, as a diagnostic tool is largely favorable, they are far from perfect. For instance, they cannot distinguish between acute fear and chronic anxiety [29]. Additionally, salivary cortisol concentrations reflect free serum cortisol concentrations, which is the unbound and biologically active 5% of total cortisol under basal conditions. On the other hand, measuring plasma cortisol concentration yields total cortisol. In other words, salivary cortisol can only be a surrogate for free serum cortisol concentrations [30]. Another study demonstrated that the validity of assessing manganese exposure through saliva samples needs further study. The correlation between manganese exposure in water and salivary levels is the weakest of the candidate biomarkers, including saliva, hair, and toenails [31].

### 5.2. Proteomics

Soluble salivary immune mediators such as C-reactive protein (CRP), interleukin-6 (IL-6), IL-1beta, IL-8, tumor necrosis factor-alpha (TNF-alpha), and secretory immunoglobin A (s-IgA), can be measured in salivary samples and serve as markers for systemic inflammation, including gingival inflammation [32]. In the case of children and youth with Obsessive-Compulsive Disorder (OCD), the elevated salivary levels of lysozyme, alpha-amylase, secretory s-IgA, CRP, IL-6, IL-1beta, and TNF-alpha are found to be associated with OCD diagnosis and symptom severity [32,33]. Melatonin secretion can also be detected and collected in saliva to evaluate one’s sleep [34], with the possibility of an at-home collection [35].

### 5.3. Genomics

Salivary extracellular vesicles (EVs) are reliable diagnostic biomarkers for detecting mild traumatic brain injury (TBI). Genetic profiling with the real-time PCR of salivary EVs showed that more than 50 genes, and more significantly three of them (CDC2, CSNK1A1, and CTSD), are upregulated in emergency department patients and in concussion clinic patients, compared to control groups (*p* < 0.05) **[36]**. Salivary DNA samples can also help researchers determine epigenetic changes in maltreated school-aged low-income children. The increase or the decrease in methylation varies depending on the sex of the child and the timing of maltreatment. In general, the mean difference in methylation between maltreated children and the control group was 6.2%. Epigenetic modifications are also seen in the glucocorticoid receptor gene (nuclear receptor subfamily 3, group C, member 1 genes), dopaminergic gene (ankyrin repeat and kinase domain containing 1) and alcohol-metabolizing gene (aldehyde dehydrogenase 2), which are particularly important for the development of psychopathology in maltreated children [37].

### 5.4. Transcriptomics

MicroRNA (miRNA) expression in the saliva is shown to be an accurate biomarker for traumatic brain injury (TBI) in Hicks’ study. The 2018 article published in the Journal of Neurotrauma compared changes in miRNA concentration after childhood TBI in CSF and in saliva. A total of 214 miRNAs were detected in CSF, where 63% of them were also present in saliva, and 10% had parallel changes in both saliva and CSF. Six miRNAs had similar changes in both CSF and saliva: miR-182-5p, miR-221-3p, mir-26b-5p, miR-320c, miR-29c-3p, and miR-30e-5p; three of them were related to neuronal development. Attention difficulty reported by the child and parent was directly correlated with the concentration of miR-320c [25].

Another study explored the relationship between miRNA expression in saliva and detecting prolonged concussion symptoms [38]. Researchers could accurately identify patients with prolonged symptoms using levels of 5 miRNAs (miR-320c-1, miR-133a-5p, miR-769-5p, let-7a-3p, and miR-1307-3p) and logistic regression (area under the curve, 0.856; 95% CI, 0.822–0.890). One month after injury, the expression of three miRNAs was associated with specific symptoms: miR-320c-1 was associated with memory difficulty; miR-629 with headaches; and let-7b-5p with fatigue. Similarly, a study compared salivary miRNA expression in two groups of children: those with persistent post-concussive symptoms (PPCS); and those without PPCS, at three different time points (within one week of injury, one to two weeks post-injury and four weeks post-injury) [39]. The study analyzed a total of 827 miRNAs, 91 of which had higher expression levels than the calculated background threshold and were included in the differential gene expression analysis. Among these 91 miRNAs, 13 had significantly different expression levels in children with PPCS at all the time points (Table 3).

### 5.5. Microbiomics

A study measured the number of EBV copies shed in saliva and assessed depressive symptoms using the Center for Epidemiologic Studies-Depression scale in female and male adolescents aged 11–17 years. Their study showed that the presence of salivary shedding of reactivated EBV is linked to depressive symptoms in female adolescents only. In fact, the interaction between sex and depressive symptoms with EBV reactivation was statistically significant (*p* < 0.01) [40].

## 6. Salivary Biomarkers as Diagnostic Tools in Metabolic, Gastrointestinal, and Pulmonary Diseases

### 6.1. Metabolomics

A pilot cohort study revealed that hepatic steatosis, or non-alcoholic fatty liver disease (NAFLD)—an important feature linked to metabolic syndrome—can be detected by elevated salivary uric acid levels in children with obesity. Saliva was sampled using a non-invasive swab collection device and analyzed using gas chromatography-mass spectrometry. Moreover, salivary uric acid increased was associated with elevated homeostatic model assessment-insulin resistance values. Despite the small sample, its findings revealed the hopeful use of salivary markers as a screening and preventive tool for hepato-metabolic comorbidities, including hepatic steatosis, with high sensitivity, specificity, and accuracy among children with obesity [41].

### 6.2. Proteomics

Studies have confirmed a significant increase in CRP and insulin concentrations in the saliva of children with obesity. An observational study in Spain followed a cohort of children from ages 8 to 12 and measured the levels of circulating inflammatory cytokines to detect the signs of obesity-related inflammation and glucose intolerance, a predisposing factor responsible for the progression of diabetes and metabolic syndrome. Increased salivary CRP, leptin, and insulin levels in assays detected differences in the body-mass index, dietary characteristics, and physical activity levels in a sex-dependent manner [42]. A large cohort study constructed and evaluated a continuous approach to a scoring system for metabolic syndrome risk factors adapted to children. The diagnostic tool proposed by Shi et al. incorporates a combination of clinical parameters, such as waist circumference and systolic blood pressure. It uses salivary measures of fasting glucose and high-density lipoprotein cholesterol as surrogates of plasma measures. In addition to finding correlations between obesity and the salivary measures of insulin, CRP, and adiponectin, the progressive nature of their suggested scoring system, as opposed to the binary definition of the disease in adults, revealed a higher level of sensitivity for pediatric populations [43].

Evaluating systemic changes in saliva can also be a potential tool for diagnosing, predicting, and monitoring pulmonary diseases in pediatrics. Like obesity, salivary CRP is suggested to be a predictor of pediatric acute respiratory illness, more importantly in pediatric pneumonia. The clinical utility of salivary CRP can be seen in an acute pediatric setting and helps predict serum CRP above 100 mg/L with high specificity. This can replace the invasive use of venipuncture in young outpatients [44]. Finally, salivary biosensors can detect early childhood asthma and predict asthma exacerbations. In their analytical cross-sectional study of children aged 6 to 12, Zamora-Mendoza et al. proposed a non-invasive method based on immunoassay and surface-enhanced Raman spectroscopy. The study identified the proteomic profile of bronchial inflammation, including elevated levels of IL-8, IL-10, and sCD163 [45]. Another small cohort study conducted by Okazaki et al. revealed that salivary surfactant protein D (SP-D) in children with asthma using ELISA was much higher than in healthy controls. This reflected the degree of bronchial inflammation in response to allergen exposure causing airway resistance and correlated with the increased indices measured by the forced oscillation technique. Increased salivary SP-D levels were associated with a higher degree of asthma exacerbation [46].

In the field of liver diseases, proteomic methodologies were also applied to look for a non-invasive method of monitoring. With the salivary cytokine profile using ELISA quantification, Davidovich et al. were able to reflect a similar pattern of serum inflammatory parameters describing the immunosuppressive status in liver-transplanted children undergoing tacrolimus therapy. The potential markers of inflammation in saliva using proteomics were identified, such as the reduced levels of IL-6 and IL-10 and the elevated levels of IL-1b [47].

### 6.3. Microbiomics

Two studies examined oral microbiota dysbiosis using bacterial 16S rRNA gene sequence analysis in children with various liver diseases. Iwasawa et al. showed that subjects with Pediatric-Onset Primary Sclerosing Cholangitis (PSC) presented significantly decreased Haemophilus and increased Oribacterium colonies in their saliva, compared to healthy participants to patients with Ulcerative Colitis (UC). By observing significant differences between PSC and UC salivary bacterial profiles, microbiome markers in the saliva can help distinguish these two diseases and provide accurate diagnosis in a non-invasive manner [48]. Another cohort study performed microbial profiling from oral swabs in children diagnosed with non-alcoholic fatty liver disease. Systemic inflammation, cirrhosis, and advanced liver disease progression were associated with increased levels of oral *Veillonella* and *Prevotella* [49]; however, further microbial profiling is needed with larger heterogeneous sample sizes, as mentioned previously (Table 4).

## 7. Salivary Biomarkers as Diagnostic Tools in Oral Diseases

In clinical practice, caries, gingivitis, and periodontitis diagnosis is mostly based on clinical, visual, and radiological findings. The following section overviews research where various proteins, microbes, and metabolites, were associated with childhood caries or periodontal disease. This research can be a basis for biochemical diagnostic tools in dental settings. Adopting such a biochemical diagnosis can improve treatment outcomes by enabling dentists to tailor therapeutic interventions to a patient’s specific biochemical needs (Table 5).

### 7.1. Metabolomics

In the case of dental caries, one study by Alqaderi et al. associated a later bedtime in Kuwaiti children with an increased incidence of caries [50]. To explain this association, the authors highlight that a later bedtime leads to changes in hormonal levels, notably a lower leptin level and a higher ghrelin level. This hormonal change leads to more frequent, carbohydrate-rich snacking in children, thus predisposing them to caries. This observation opens the door for cost-efficient, easy-to-implement sleep interventions in susceptible children to reduce their caries risk. Additionally, these hormonal changes can be detected relatively simply and non-invasively in saliva. Another study by Syed et al. associated a lower level of salivary nitric oxide (NO) and its metabolites with an increased risk of caries in children. This association can be explained by the antimicrobial role of NO in protection against caries [51]. Thus, screening children for salivary NO levels enables dentists to identify those at high-risk for caries, and tailor a dietary intervention with relatively easier compliance to raise their NO levels, and thus protect them from caries.

As for gingivitis and periodontal disease, Goodson et al. found an association between dietary phosphate intake and gingivitis in children. Indeed, phosphate acts as a local pro-inflammatory in the gingiva by reducing IL-4 production and raising that of IL-1b [58]. Thus, by screening children for salivary phosphate levels, we can identify those at high-risk for gingivitis and intervene by reducing their dietary intake to protect them from caries. Lastly, a study by Sridharan et al., focusing on periodontal disease in children with Type I Diabetes Mellitus (T1DM), observed that children with a higher alkaline phosphatase level had a greater gingival index and pocket pocking depth [59]. Thus, evaluating the salivary level of alkaline phosphatase in children with T1DM enables dentists to intervene early to avert the course of the disease and improve the children’s outcomes.

### 7.2. Proteomics

This sub-section will also focus only on caries lesions. A study by de-Sousa et al. [52] found an association between the activity of carbonic anhydrase-VI (CA-VI) and caries incidence in children. Indeed, a lower activity level of salivary CA-VI predisposes children to caries and can thus be used as a prognostic factor for caries development. Another work by Jurczak et al. associated higher levels of salivary b-defensin-2 and histatin 5, two proteins with antimicrobial properties, with more advanced carious lesions [53]. Higher levels of 1β, IL-6, IL-8, IL-10, and TNF-a are seen to be increased in gingivitis and caries-prone children [54,55]. Lastly, while this does not constitute a salivary biomarker, Hertel et al. observed a higher level of Mucin 5b, Mucin 7, and IgA, in the pellicle of caries-active children compared to their caries-free counterparts [60].

While the authors of the studies above recognize a limitation in their work, namely their small sample sizes, they encourage the pursuit of larger-scale trials to confirm their observed associations. Much like we would test gene panels to identify a defective gene in an individual, to diagnose and prognose a suspected genetic disorder, one could do a “salivary biomarker screen”, which would include the various metabolites, microbes, and proteins associated with oral disease. Such a screening tool would enable dentists to pinpoint a patient’s biochemical risk factors for oral disease and develop individualized treatment plans for their patients.

### 7.3. Microbiomics

This sub-section focuses exclusively on childhood caries. One study by Kim et al. observed that caries-free Korean children had greater diversity in their oral microbiota than their caries-active counterparts [61]. Additionally, children with caries had greater proportions of bacteria that exhibit pro-caries behavior, such as *Streptococcus* and *Granulicatella*, which invade epithelial cells, as well as *Staphylococcus* and *Veillonella*, which are involved in D-alanine metabolism. In contrast, caries-free children had greater proportions of bacteria that antagonize caries formation, notably *Neisseria* and *Lautropia*, which are involved in linoleic acid metabolism, and *Capnocytophaga*, which is involved in flavonoid biosynthesis. Another study by Hemadi et al. observed higher salivary levels of *Streptococcus mutans spp.*, *C. albicans,* and *Prevotella* spp. in children with caries, compared to their caries-free counterparts [56]. Lastly, Manzoor et al. observed higher levels of sugar fermenters, such as *Paludibacter* and *Labrenzia*, in children with caries [57].

## 8. Current Status and Advances in Saliva-Based Biosensors

**Figure 2:**
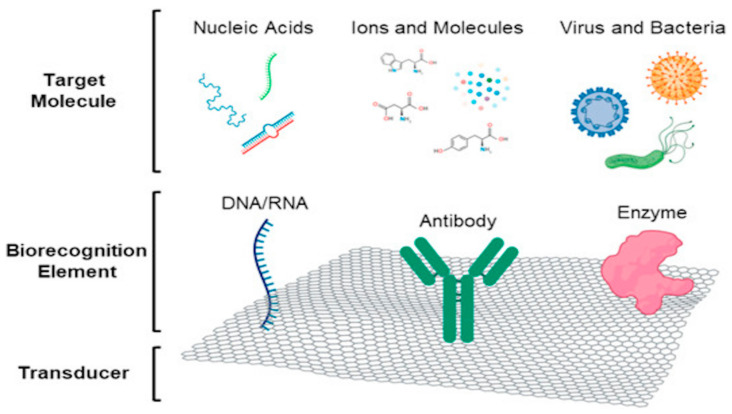
A typical saliva-based biosensor. Briefly, it consists principally of three components, a surface (carbon-based or metal) to accommodate the biorecognition element (probes, antibodies, or enzymes), which captures the target (genomic material, antibodies, proteins, etc.). The molecular interactions induce colorimetric or spectrometric changes, which can be observed with their corresponding detection technologies. Image used with permission from Goldoni et al., 2021 [63].

The importance of saliva as a diagnostic tool came into light during the advent of the COVID-19 pandemic. Its non-invasiveness and the ease of collection ensured the safety of healthcare workers; nonetheless, nasopharyngeal swabs remained the gold standard. It evidenced how saliva can become an essential part of diagnostic procedures. Antibody antigen-based reactions can be efficiently utilized and detected by colorimetric or spectrophotometric changes. A prototype for a saliva-based MMP8 detector using specific antibodies and surface acoustic wave (SAW) technology was developed for periodontal disease and was seen to have comparable sensitivity and specificity to the standard ELISA-based methods [62] (Figure 2).

Stefan-van Staden et al. proposed a new method of assay of estradiol (E2), testosterone (T2), and dihydrotestosterone (DHT) based on stochastic microsensors that allow the earlier detection and prevention of hormonal, metabolic, and developmental problems in children. This could replace the classical measurement of electrochemical markers using ELISA or liquid chromatography-tandem mass spectrometry. The biosensor is designed using diamond paste as the matrix and three different electroactive materials: maltodextrin, a-cyclodextrin, and 5,10,15,20-tetraphenyl-21H,23H porphyrin [64]. The response to stochastic microsensors is based on channel conductivity, which is the degree of alteration of a current traversing a channel under a controlled potential of 125 mV when a steroidal hormone binds onto the channel wall. By manipulating the channel conductivity of analytes, stochastic sensors are described as having high reliability and selectivity, which could overcome the limitations of the standard method, such as its insufficient sensitivity for children’s saliva. The results demonstrated that, compared to the standard method using ELISA, the stochastic biosensor could perform reliable pattern recognition, identification, and the quantification of the three hormones at very low concentrations in saliva samples [64].

Lukose et al. also highlighted optical technologies using photonics to rapidly screen abnormal health conditions and viral infections rapidly and universally through saliva. The article explored optical spectroscopy techniques that more precisely involve the UV-visible-infrared region, including surface plasmon resonance, Raman, IR spectroscopy, and laser-induced fluorescence [65]. For instance, a previous section of our paper discussed the potential use of surface-enhanced Raman spectroscopy to detect the proteomic characteristics of asthma in children. Non-invasive, photonic-based biosensors constitute a hopeful field of new diagnostic tools due to their capacity to provide the remote, contactless evaluation and identification of conditions, including communicable diseases such as COVID-19. In addition to detecting viral, bacterial, and parasitical infections, photonics can be applied in a wide range of health conditions, such as various cancers, oral diseases, metabolic and systemic diseases, as well as the detection of drug-related biomarkers in saliva [65]. Finally, Lukose and al. suggested the possibility of a multi-modal approach in which a combination of photonics tools, accompanied by artificial intelligence, could further optimize the specificity and sensitivity of these promising biosensors.

## 9. Conclusions

We have reviewed several methodologies that performed with good sensitivity and specificity, as well as additional advantages covering some of the important limitations of standard diagnostic tests performed today in the pediatric population. Acknowledging its non-invasiveness and ubiquitous applications, saliva as a probing biofluid sample remains highly attractive. It requires further investigations with larger samples and better analytical and comparative evidence. The pursuit of further studies on these biomarkers can be highly impactful. Indeed, salivary diagnostic/prognostic tools are less invasive and less harmful than current tools and enable physicians to intervene early, altering the course of the disease and significantly reducing suffering and disability in patients.

While sensitivity and specificity to the target molecules are satisfactory in the studies explored, the specificity to a particular condition remains a challenge. For example, pro-inflammatory markers can be commonly identified in autoimmune disorders, stress, poor oral health, and pulmonary conditions. Identifying a marker highly specific to each condition is imperative. Nonetheless, diagnosing infectious diseases like COVID-19, and periodontal diseases, using microbiome-specific screens, specific proteins in ASD, and miRNAs in various systemic conditions, are promising as salivary biosensor applications.

## Figures and Tables

**Table 1:** Investigated autoimmune disorders and their respective biomarkers.

	Disease	Observed Biomarker	Methodology	Ref.
1.	Type I Diabetes Mellitus	Oxidative Stress Index (OSI), assessed by examining: - Total Antioxidant Status (TAS) - Total Oxidant Status (TOS)	Commercially available assay kits (Rel Assay, Mega Tıp, Gaziantep, Turkey)	[1]
2.	Juvenile Idiopathic Arthritis	TNF-alpha, TNFRSF1B, MMP-1, MMP-2, MMP-3, MMP-13, IL-1alpha, IL-1beta, IL-1 RII, IL-2, IL-6, IL-6Ralpha, IL-8, IL-10, IL-12, CCL2, CCL3, CCL11, CCL22, CXCL9, S100A8	Customized R&D-bead based immunoassay (R&D SYSTEMS/Bio-Techne; Minneapolis, MN, USA)	[2]
3.	Pediatric-Onset Multiple Sclerosis	FLC as Monomers & Dimers (kapapaM, lambdaM, kappaD, lambdaD)	Gel Analysis & Blotting using rabbit antibodies to human Ig kappa and lambda light chains	[3]
4.	Sjogren’s Syndrome	IL27, MIA, CCL4, TNFRSF18 and TNF-alpha (Among others)	Multiplex fluorescent microparticle-based immunoassays	[4]

**Table 2:** Most commonly recurring developmental conditions and their respective biomarkers.

	Disease	Observed Biomarker	Methodology	Ref.
1.	Autism Spectrum Disorder	Salivary zinc	Plasma emission spectroscopy	[8]
		16s rRNA	16s RNA gene amplicon sequencing	[23,24]
		Calmodulin-3 Plastin-2 S100-a7	Bio-Rad protein assay	[17]
2.	Skeletal Maturity	LDH	Spectrophotometry	[5]
		ALP	ALP and protein assay	[6]
		Salivary B-ALP	ELISA	[7]
		IGF-1 IGFBP-3	Spectrophotometry	[10,11]
3.	Attention Deficit Hyperactivity Disorder	DHEA CpG methylation	TaqMan assay	[22]

**Table 3:** Neuropsychiatric conditions and their respective biomarkers.

	Condition	Observed Biomarker	Methodology	Ref.
1.	**Stress**	s-IgA	s-IgA ELISA kit (EUROIMMUN AG; Luebeck, Germany)	[27]
		Cortisol	Commercial ELISA kit (Salimetrics, USA)	[27]
		Alpha-amylase	Commercially available chemoimmunoluminiscence assay kits (Cobas integra400 plus, Roche Diagnostics, Risch-Rotkreuz, Switzerland) and analyzer (Cobas e411, Roche Diagnostics, Risch-Rotkreuz, Switzerland)	[29]
2.	**Cerebral Palsy**	IL-1β IL-6 IL-8 IL-10 TNF-α	CBA Cytokine Inflammatory Kit (Becton Dickinson, CA, USA)	[28]
3.	**Systemic Inflammation**	C-reactive proteins	Commercially available enzyme-linked immunoassay kit from Salimetrics, Suffolk, UK	[32]
		Cytokine concentrations (IL-1β, IL-6, IL-8, and TNF-α)	MILLIPLEX MAP HCYTOMAG-60K-04 kit (Millipore, Billerica, MA, USA)	[31]
		s-IgA	Colorimetric immunoenzymatic assay (ELISA) using an s-IgA saliva kit (DiaMetra, Milano, Italy)	[31]
4.	**Mild TBI**	CDC2, CSNK1A1 and CTSD	NanoSight NS500 instrument (Nanosight, Malvern, UK), transmission electron microscopy, Western blot analysis, Taqman	[36]
		miR-182-5p, miR-221-3p, mir-26b-5p, miR-320c, miR-29c-3p, miR-30e-5p	Oragene RE-100 saliva collection kit (DNA Genotek; Ottawa, Canada)	[25]
5.	**Child Maltreatment**	ALDH2, ANKK1 and NR3C1	Oragene DNA Self-Collection kits	[37]
6.	**Prolonged Concussion Syndrome**	miR-320c-1, miR-133a-5p, miR-769-5p, let-7a-3p, and miR-1307-3p	Plasma/Serum Circulating and Exosomal RNA Purification Kits (Norgen Biotek)	[38,40]
		hsa-miR-95-3p, hsa-miR-301a-5p, hsa-miR-626, hsa-miR-548y, hsa-miR-203a-5p, hsa-miR-548e-5p, hsa-miR-585-3p, hsa-miR-378h, hsa-miR-1323, hsa-miR-183-5p, hsa-miR-200a-3p, hsa-miR-888-5p, hsa-miR-199a-3p + hsa-miR-199b-3p	nCounter^®^ human V3 miRNA assay kit (NanoString Technologies Inc., Seattle, WA, USA)	[39]
7.	**Depression**	EBV	QiaAmp blood kit (Qiagen, Valencia, CA, USA)	[40]

**Table 4:** Common metabolic, gastrointestinal, and pulmonary conditions and their respective biomarkers.

	Condition	Observed Biomarker	Methodology	Ref.
1.	**Non-Alcoholic Fatty Liver Disease**	Uric acid	Gas chromatography-mass spectrometry	[41]
		*Veillonella* colonies *Prevotella* colonies	Bacterial 16S rRNA gene sequencing	[49]
2.	**Liver Graft versus Host Rejection**	IL-6 IL-10 IL-1b	ELISA	[47]
3.	**Primary Sclerosing Cholangitis**	*Haemophilus* colonies *Oribacterium* colonies	Bacterial 16S rRNA gene sequencing	[48]
4.	**Obesity**	Insulin	Multiplex magnetic bead assays	[42]
		CRP		[43]
		Leptin		
		Adiponectin		
5.	**Pediatric Pneumonia**	CRP	ELISA	[44]
		CP-D	ELISA	[46]
6.	**Asthma**	IL-8 IL-10 sCD163	Immunoassay and surface-enhanced Raman spectroscopy	[45]

**Table 5:** Oral diseases and their respective studied biomarkers.

	Disease	Observed Biomarker	Methodology	Ref.
1.	**Dental Caries**	Leptin and Ghrelin	Multiplex Magnetic Bead Panels on a Luminex 200 Platform	[50]
		NO (measured as total nitrates and nitrites)	Griess reaction method	[51]
		Carbonic Anhydrase-VI	Zymography method	[52]
		Beta-defensin-2 Histatin-5	ELISA	[53]
		IL6 IL8 TNF-alpha	ELISA	[54,55]
		*Streptococcus mutans* spp. *C. albicans* *Paludibacter* *Neisseria* (Among others)	16S rRNA	[56,57]
2.	**Periodontal Disease**	Phosphate	Multiplex Magnetic Bead Panels	[58]
		Alkaline Phosphatase (In T1DM patients only)	DEA-AMP Method	[59]

## Data Availability

Not applicable.
